# Quantitative shotgun proteomics distinguishes wound-healing biomarker signatures in common carp skin mucus in response to *Ichthyophthirius multifiliis*

**DOI:** 10.1186/s13567-018-0535-9

**Published:** 2018-04-20

**Authors:** Mona Saleh, Gokhlesh Kumar, Abdel-Azeem Abdel-Baki, Mohamed A. Dkhil, Mansour El-Matbouli, Saleh Al-Quraishy

**Affiliations:** 1 Clinical Division of Fish Medicine, University of Veterinary Medicine, Vienna, Austria; 2 Zoology Department, College of Science, King Saud University, Riyadh, Saudi Arabia; 3 Zoology Department, Faculty of Science, Beni-Suef University, Beni Suef, Egypt; 4 Department of Zoology and Entomology, Faculty of Science, Helwan University, Cairo, Egypt

## Abstract

*Ichthyophthirius multifiliis* is a ciliated protozoan parasite recognized as one of the most pathogenic diseases of wild and cultured freshwater fish. Fish skin mucus plays a significant role against invading pathogens. However, the protein-based modulation against infection with *I. multifiliis*, of host fish at this barrier is unknown. Thus, we investigated the skin mucus proteome of common carp using a shotgun proteomic approach at days 1 and 9 after *I. multifiliis* exposure. We identified 25 differentially expressed proteins in infected carp skin mucus. Upregulated proteins were mainly involved in metabolism, whereas downregulated proteins were mainly structural. This is the first proteomic analysis of infected common carp skin mucus, and it provides novel information about proteome alteration caused by *I. multifiliis*. Furthermore, we identified novel proteins with yet unknown function in common carp following penetrating injuries such as olfactomedin 4, lumican, dermatopontin, papilin and I cytoskeletal 18. This analysis, therefore, represents a key for the search for potential biomarkers, which can help in a better understanding and monitoring of interactions between carp and *I. multifiliis*. This proteomic study not only provides information on the protein-level pathways involved in fish-ciliate interactions but also could represent a complementary system for studying tissue repair.

**Electronic supplementary material:**

The online version of this article (10.1186/s13567-018-0535-9) contains supplementary material, which is available to authorized users.

## Introduction

Intensive fish farming raises the incidence of injuries and diseases. Skin mucus acts as a mechanical, physical, chemical, biological, and immunological barrier against any external stressor [1]. Skin mucus has become a novel area of research and a true indicator of the immune status of fish. The current advances in proteomics studies have been used for the characterization, identification, and quantification of proteins [2]. The skin mucus proteome has recently been studied in several fish species. These studies have revealed novel molecules involved in protection and immunity of this mucosal surface. Furthermore, the modulation of the skin mucus proteome has been investigated in response to infection, wounds, stress, or after administration of different dietary supplements [3–7].

Wound healing and tissue repair are highly complex and indispensable processes to ensure the survival and health of an organism. The regenerative competence differs considerably across organs and organisms and requires the harmonized interaction of different cell types, signaling systems including cytokines, growth factors, cellular matrix molecules, and different classes of proteases, as well as their inhibitors [8]. However, the information on cellular and molecular mechanisms involved in these processes is still limited.

Certain lower vertebrates such as teleost fish have greater regenerative capacity than mammals. Therefore, they are used as a model for tissue regeneration in humans. Cordero et al. [1] investigated the modification of the skin mucus proteome after inducing chronic wounds in gilthead seabream (*Sparus aurata*) and found that structural proteins, which are involved in tissue repair, were downregulated. However, little information is available on the proteomic modification of fish skin mucus in response to tissue damage caused by invading pathogens.

Gonzalez et al. [9] reported a recruitment of neutrophils along with an initial upregulation, followed by the downregulation of the proinflammatory cytokines (IL1β) and the chemokine receptor (CXCR1) in *Ichthyophthirius multifiliis*-infected common carp skin, whereas Jørgensen [10] directly visualized the recruitment of neutrophils to infected areas in the skin. This is comparable to the gene expression pattern induced by mechanical injuries and attributed to *I. multifiliis*-induced injuries (penetrating wounds) at infection sites [9].

We hypothesized that a proteomic evaluation of skin mucus from non-exposed fish against those exposed to *I. multifiliis* would facilitate in identifying specific mucus components that are involved in carp (*Cyprinus Carpio*) immune response to tissue damage caused by the parasite. The aim of this study was to investigate the modulation of the skin mucus after infection with *I. multifiliis* using quantitative proteomics to provide insights into the post-transcriptional and post-translational regulation of skin mucus proteins.

## Materials and methods

### Animals and collection of samples

Specific pathogen-free common carp (11 ± 1 cm) were obtained from a certified Austrian hatchery and acclimatized for 2 weeks under controlled laboratory conditions at 16 °C. The fish were fed 1% body weight per day using a commercial pellet diet (Garant Aqua, Pöchlarn, Austria). Before infection, 36 fish were distributed between six aquaria, six fish per aquarium. There were two groups: exposed and non-exposed control. To mimic natural exposure [11], the fish were exposed to *I. multifiliis* by cohabitation with naturally infected giant gourami (*Osphronemus goramy*) obtained from a pet store. The giant gourami did not show any other ectoparasite or signs of a secondary bacterial infection. The gouramis were also certified as free from *Aphanomyces invadans*, the causative agent of the epizootic ulcerative syndrome and the Epizootic Haematopoietic Necrosis Virus, however, it should be kept in mind that they could harbour another disease. At 1 and 9 days post-exposure (dpe), common carp (*n* = 3) from each of the exposed and non-exposed control groups were anesthetized using 100 mg/L of ethyl 3-aminobenzoate methanesulfonate (MS-222; Sigma, Darmstadt, Germany). Using sterile glass slides, mucus was collected from the fish skin while avoiding blood contamination and excluding the ventral body surface close to the anal pore to prevent fecal contamination. Collected mucus was transferred into 1.5-mL microcentrifuge tubes, directly placed on ice, and then stored in a −80 °C freezer until further analysis.

### Protein extraction

Fish mucus was solubilized using 400 µL pre-cooled denaturing lysis buffer (7 M urea, 2 M thiourea, 4% CHAPS, and 1% DTT) containing mammalian protease inhibitor cocktail (Sigma Aldrich, Vienna, Austria). Fish mucus suspensions were disrupted by sonication. The lysates were then incubated overnight at 4 °C. Subsequently, the lysates were vortexed, and then centrifuged at 18 000 × *g* for 30 min at 4 °C and the supernatants were collected. The total protein concentration of each lysate was determined colorimetrically with a NanoDrop 2000c (Thermo Fisher Scientific, USA) spectrophotometer using a Pierce 660 nm Protein Assay (Pierce, Thermo Fisher Scientific) according to the manufacturer’s instructions.

### Protein separation

The protein samples (40 µg per lane) in biological and technical triplicate were subjected to electrophoresis in 10% polyacrylamide separating gels. After electrophoresis, the gels were stained with silver staining.

### In-gel digestion

Protein bands were excised manually from silver-stained one-dimensional gels. After washing and destaining, bands were reduced with dithiothreitol and alkylated with iodoacetamide [12]. In-gel digestion was performed using trypsin (Trypsin Gold, Mass Spectrometry Grade, Promega, Madison, WI, USA) with a final trypsin concentration of 20 ng/µL in 50 mM aqueous ammonium bicarbonate and 5 mM CaCl_2_. In-gel digestion was performed for 8 h at 37 °C using trypsin at a final concentration of 20 ng/µL in 50 mM aqueous ammonium bicarbonate and 5 mM CaCl_2_ [13]. Subsequently, peptides were extracted with three changes of 30 µL of 5% trifluoroacetic (TFA) acid in 50% aqueous acetonitrile supported by ultrasonication for 10 min per change. Extracted peptides were dried down in a vacuum concentrator (Eppendorf, Hamburg, Germany), and then re-dissolved in 0.1% aqueous TFA before liquid chromatography–mass spectrometry (LC–MS) injection.

### Liquid chromatography–tandom mass spectrometry (LC–MS/MS) analysis

Peptides were separated on a nano-HPLC Ultimate 3000 RSLC system (Dionex, USA). The samples were pre-concentrated and desalted using a 5-mm Acclaim PepMap μ-Precolumn (300 µm inner diameter, 5 µm particle size, and 100 Å pore size; Dionex, USA). For sample loading and desalting, 2% ACN in ultra-pure H_2_O with 0.05% TFA was used as a mobile phase with a flow rate of 5 µL/min. Peptides were separated on a 25-cm Acclaim PepMap C18 column (75 µm inner diameter, 2 µm particle size, and 100 Å pore size) with a flow rate of 300 nL/min. The gradient started with 4% B (80% ACN with 0.1% formic acid) and increased to 35% B in 120 min. A washing step with 90% B was then performed. Mobile phase A contained ultra-pure H_2_O with 0.1% formic acid.

### Quadrupole time of flight (QTOF) mass spectrometry for sequential window acquisition of all theoretical mass spectra (SWATH) measurements

For mass spectrometric analysis, the LC was coupled to a high-resolution quadrupole time of flight mass spectrometer (Triple TOF 5600+, Sciex, USA). Data-independent Sequential Window Acquisition of all Theoretical spectra (SWATH runs) technology based on MS2 quantification was used for quantitative measurements [14]. Peptides were fragmented in 35 fixed fragmentation windows of 20 Da in the range of 400–1100 Da with an accumulation time of 50 min in TOF–MS and 80 min in product ion mode. The nano-HPLC system was operated by Chromeleon 6.8 (Dionex, USA) and the MS by Analyst Software 1.6 (Sciex, USA).

### Data processing, quantification, and statistical evaluation

Acquired raw data were assessed using ProteinPilot software version 5.0 (Sciex, USA) for re-calibration and database searches using NCBI entries of *Cyprinus* (taxonomy id: 7961). Mass tolerance in MS mode was set with 0.05 and 0.1 Da in MSMS mode for the rapid re-calibration search, and 0.0011 Da in MS and 0.01 Da in MSMS mode for the final search. The following sample parameters were used trypsin digestion, cysteine alkylation set to iodoacetamide, and the processing parameter was set to a rapid ID search effort. False discovery rate analysis (FDR) was evaluated using the integrated tools in ProteinPilot and was set to < 1% at the protein level. The results of Information Dependent Acquisition (IDA) identification were used to create the SWATH ion library with the MS/MS (ALL) with SWATH Acquisition MicroApp 2.0 in PeakView 2.2 (both Sciex, USA). Peptides were chosen based on an FDR rate < 1%, excluding shared and modified peptides. Up to six peptides per protein and up to six transitions per peptide were used. MarkerView 1.2.1 (Sciex) was used for the calculation of peak areas of SWATH samples after retention time alignment and normalization using total area sums. The resulting protein lists were then used for the visualization of data after principal component analysis in the form of loading and score plots to get the first impression of the overall data structure and assess the variability between technical and biological replicates.

To determine differentially expressed proteins in the mucus samples, statistical evaluation was performed in R programming language [15]. The differential expression of proteins was evaluated using one-way analysis of variance (ANOVA) for each protein. For multiple testing, the method of Benjamini and Hochberg [16] was used to control the FDR. The differences were considered significant if adjusted *p* values were smaller than the significance level of *α* = 0.01. For such proteins, the honest significant difference method of Tukey was applied as post hoc test to assess the significance of the pairwise comparisons. Protein expression was considered differential if the adjusted *p* value was below *α* and the absolute fold change was at least three (fold change < −3 or > +3).

PANTHER classification system was used for the classification of biological processes, cellular components, and molecular functions of differentially expressed proteins. To determine the network of differentially expressed proteins, amino acid sequences of identified proteins were BLAST searched using the string tool based on the study conducted on *Danio rerio* [17]. The representation of the protein–protein network was analyzed at the confidence score of 0.15 in the database, experiment, text mining, co-expression, neighborhood, gene fusion, and co-occurrence database.

## Results

### Protein identification

**Figure 1:**
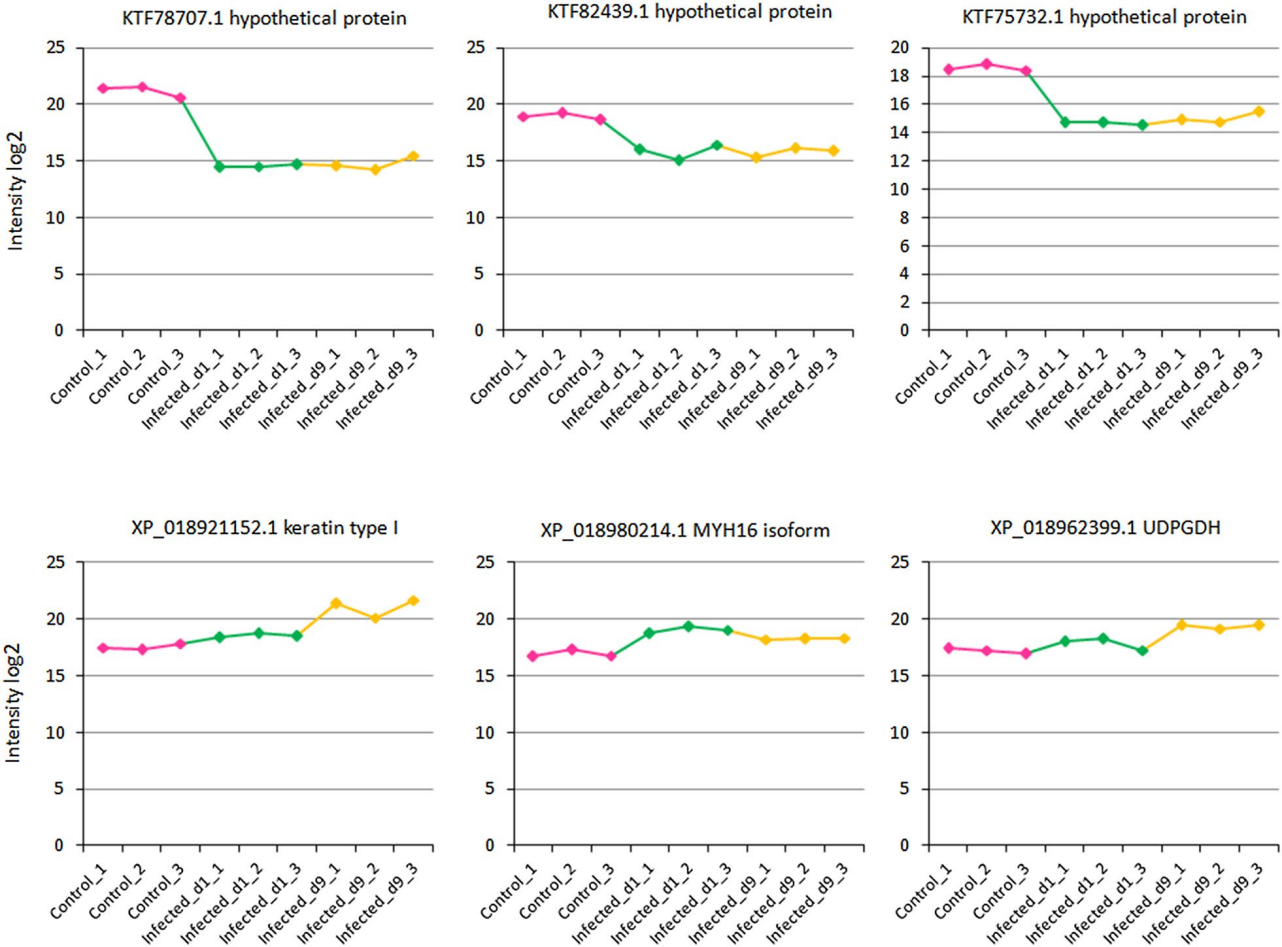
**Expression plots of top candidate carp skin mucus proteins.** Plots show proteins up and down-regulation of carp skin mucus at 0 (control), 1, and 9 days post-exposure.

We identified 1233 proteins in the skin mucus of common carp. Twenty-five structural and metabolism proteins were differentially expressed in infected carp skin mucus. Nineteen proteins were mainly structural and extracellular matrix proteins, whereas six proteins have a distinctive role in metabolism (Tables 1 and 2). These proteins were associated with e.g. cellular, metabolic, developmental and immune processes, as well as biological regulation, localization, response to a stimulus, and multicellular organismal process. Further, they were implicated in binding, catalytic, receptor, signal transducer, structural, molecular, and transporter activities. The expression of top skin mucus proteins is shown in Figure 1. The infected samples either show lower quantitative values of collagen alpha, lumican, dermatopontin, and papilin or higher quantitative values of keratin type I, myosin, and UDP-glucose 6-dehydrogenase than the control samples.

**Table 1:** **Differentially expressed structural and extracellular matrix proteins of common carp**

NCBI accession number	Protein	Number of quantified peptides	BLASTp results	1 dpe	9 dpe	Function
Structural and extracellular matrix proteins						
KTG36050.1	cypCar_00022254 [*Cyprinus carpio*]	6	collagen alpha-3(VI) chain-like isoform X3 [*Salmo sala*r], XP_014019344.1, 62% identity	−18.7*	−37.1*	Matrix component organisation
KTF73577.1	cypCar_00045321, partial [*Cyprinus carpio*]	6	collagen alpha-2(I) chain-like [*Sinocyclocheilus rhinocerous*], XP_016385859.1, 90% identity	−50.2*	−35.7*	Matrix component organisation
XP_018968199.1	collagen alpha-1(I) chain-like [*Cyprinus carpio*]	5	–	−11.3*	−9.9*	Matrix component organisation
XP_018967439.1	collagen alpha-2(VI) chain-like [*Cyprinus carpio*]	3	–	−6.0*	−4.5*	Matrix component organisation
KTF78707.1	cypCar_00043888, partial [*Cyprinus carpio*]	4	–	−99.9*	−88.2*	Matrix component organisation
XP_018967802.1	collagen alpha-1(VI) chain-like [*Cyprinus carpio*]	5	–	−6.1*	−8.3*	Matrix component organisation
XP_018962933.1	collagen alpha-1(XIV) chain-like [*Cyprinus carpio*]	2	–	−27.3*	−31.2*	Matrix component organisation
KTF76685.1	cypCar_00016174 [*Cyprinus carpio*]	2	collagen alpha-2(VI) chain-like [*Cyprinus carpio*], XP_018932781.1, 96% identity	−3.7*	−5.3*	Matrix component organisation
XP_018933497.1	collagen alpha-1(XIV) chain-like [*Cyprinus carpio*]	2	–	−5.1*	−5.2*	Matrix component organisation
XP_018950001.1	dermatopontin-like [*Cyprinus carpio*]	2	–	−4.9*	−10.9*	Matrix component organisation
XP_018958339.1	src substrate cortactin-like [*Cyprinus carpio*]	2	–	−1.2	2.6	Actin regulatory protein, stabilises actin filaments
KTF82439.1	cypCar_00015496 [*Cyprinus carpio*]	2	lumican-like [*Sinocyclocheilus rhinocerous*], XP_016414194.1, 96% identity	−8.7*	−9.1*	Binds collagen fibrils and regulates its structure, and enhances macrophages and neutrophils recruitment
P_018921152.1	keratin, type I cytoskeletal 18 [*Cyprinus carpio*]	6	–	2.0	11.4*	Protect cells from mechanical and non mechanical injuries, pore-forming activities
XP_018948461.1	keratin, type I cytoskeletal 18-like [*Cyprinus carpio*]	3	–	*1.2*	−3.5*	Protect cells from mechanical and non mechanical injuries, pore-forming activities
XP_018980214.1	MYH16 isoform X1 [*Cyprinus carpio*]	6	myosin-11-like [*Sinocyclocheilus rhinocerous*] Sequence ID: XP_016405427.1, 90% identity	4.4*	2.5	Transendothelial migration of leukocytes
XP_018930066.1	LOC109057295 [*Cyprinus carpio*]	6	myosin heavy chain, clone 203 [Danio rerio], XP_009289654.1, 48% identity	−1.4	6*	Transendothelial migration of leukocytes
KTF75732.1	cypCar_00036737 [*Cyprinus carpio*]	5	PREDICTED: papilin-like [*Sinocyclocheilus anshuiensis*] XP_016354784.1, 92% identity	−15*	−11.4*	metalloproteinase inhibitor, tissue rearrangement
KTF72113.1	cypCar_00043727, partial [*Cyprinus carpio*]	2	PREDICTED: neoverrucotoxin subunit beta-like [*Sinocyclocheilus anshuiensis*], XP_016330044.1, 91% identity	*−1*	3.5*	Microtubule organization and stabilization, pore-forming activities
XP_018924262.1	olfactomedin-4-like isoform X2 [*Cyprinus carpio*]	4	–	3.3*	1.3	Negative feedback effect on NF-κB activation

Fold change (infected vs control) was statistically analyzed in *Ichthyophthirius multifiliis* exposed infected common carp at 1 and 9 days post-exposure (dpe). * Denotes values (italic) statistically significant according to both ANOVA with FDR-adjusted *p* value < 0.001 and fold change < −3 or > +3.

**Table 2:** **Differentially expressed metabolism proteins of common carp**

NCBI Accession number	Protein	Number of quantified peptides	BLASTp results	1 dpe	9 dpe	Function
Metabolism proteins						
XP_018962399.1	UDP-glucose 6-dehydrogenase [*Cyprinus carpio*]	6	–	1.6	4.4*	Catalyzes the conversion of glucose 6-phosphate to 6-phosphogluconate
XP_018964044.1	clustered mitochondria protein homolog [*Cyprinus carpio*]	2	–	1.5	3.5*	Regulate mitochondrial metabolism
XP_018973586.1	arachidonate 5-lipoxygenase-like [*Cyprinus carpio*]	4	–	−4*	−10.1*	Transforms essential fatty acid (EFA) substrates into leukotrienes
XP_018937956.1	arachidonate 5-lipoxygenase-like [*Cyprinus carpio*]	4	–	−3*	−6*	Transforms essential fatty acid (EFA) substrates into leukotrienes
XP_018936787.1	PDZ and LIM domain protein 1-like [*Cyprinus carpio*]	4	–	−1.0	3.0*	Bind to the NF-κB subunit p65 and inhibits its transcriptional activity
XP_018979180.1	relA-associated inhibitor-like [*Cyprinus carpio*]	4	–	−2.1	2.8	Bind to the NF-κB subunit p65 and inhibits its transcriptional activity

Fold change (infected vs control) was statistically analyzed in *Ichthyophthirius multifiliis* exposed infected common carp at 1 and 9 days post-exposure (dpe). * Denotes values (italic) statistically significant according to both ANOVA with FDR-adjusted *p* value < 0.001 and fold change < −3 or > +3.

### Structural and extracellular matrix proteins

We identified 11 members belonging to 6 collagen-alpha family proteins (type I alpha 1a, type I alpha 2, type VI alpha 1, type VI alpha 2, type VI alpha 3, and type XIV alpha 1). In addition, we identified the following proteins: Src substrate cortactin-like, dermatopontin-like, papilin-like, neoverrucotoxin, olfactomedin 4, lumican, myosin (*n* = 2), and keratin (*n* = 2). Most of the structural proteins were downregulated (Table 1). The extracellular matrix proteins such as olfactomedin 4, neoverrucotoxin, myosin, and keratin were upregulated. The extracellular matrix components that are hallmarks of the wound matrix [18] were identified with differential quantitative values in infected and non-infected carp. These components include collagen alpha, neoverrucotoxin (contains fibronectin type III domain), olfactomedin 4, and lumican. Collagen alpha members were extremely downregulated in infected carp. These observations link the downregulation of the collagen alpha to penetrating wounds caused by *I. multifiliis*, and lead to the hypothesis that deficiency in collagen alpha may reflect an impaired healing state during parasite attachment to carp skin.

### Metabolism proteins

We identified the following metabolism proteins (Table 2): UDP-glucose 6-dehydrogenase, clustered mitochondria protein homolog (CLUH), PDZ/LIM domain protein 1-like, RelA-associated inhibitor (RAI)-like, and arachidonate 5-lipoxygenase (ALOX5)-like (*n* = 2). The expression of these proteins was upregulated at 9 dpe, except for ALOX5-like.

### Protein–protein interaction network

**Figure 2:**
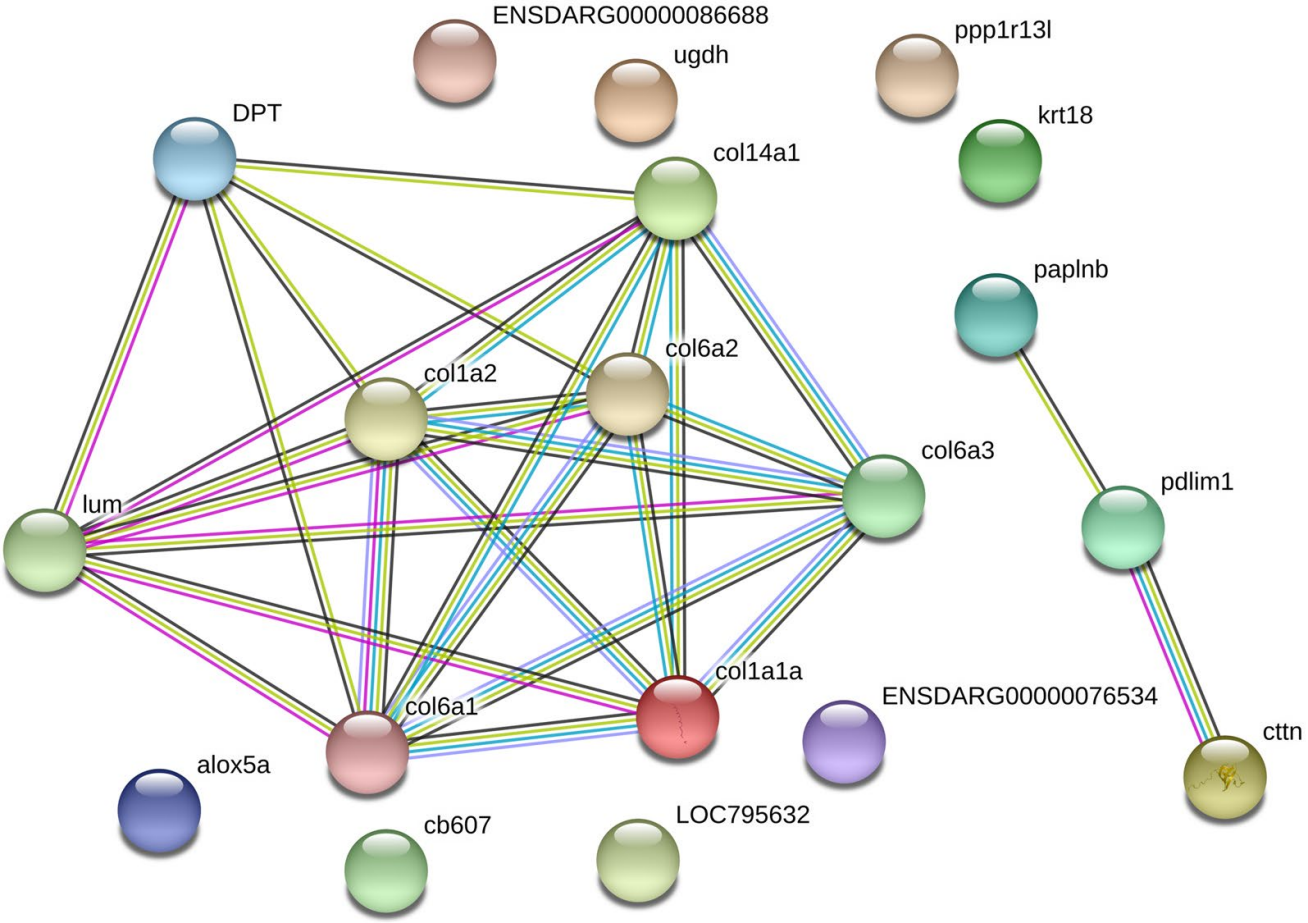
**The protein–protein interaction network of carp skin mucus proteins.** In this network, nodes are proteins, lines represent the predicted functional associations, and the number of lines represents the strength of predicted functional interactions between proteins. Eight proteins including six collagen alpha family proteins (col1a2, col1a1a, col6a1, col6a2, col6a3, col14a1), lumican (lum), and dermatopontin (DPT) were involved in the protein–protein interaction network. The figure shows that collagen type I alpha is the central node of the protein–protein interaction network.

As shown in Figure 2, eight proteins (six collagen alpha family proteins, lumican, and dermatopontin proteins) were involved in the protein–protein interaction network. Details of protein abbreviation, node colour, edge interaction, network stats and functional enrichment are described in Additional file 1. The data showed that collagen type I alpha was the central node of protein–protein interaction analysis. These differentially up- and downregulated proteins showed two KEGG pathways: extracellular matrix receptor interaction and focal adhesion. Pfam and InterPro database evaluation (as a part of the string) revealed enrichment in collagen triple helix repeat and von Willebrand factor, type A domain-containing proteins. Similarly, on the same dataset, the PANTHER analysis found an increase in the following biological processes: cellular, metabolic, biological regulation, and response to a stimulus.

## Discussion

In vertebrates, the mucosal immune system has crucial functions against infections. It prevents the uptake of microorganisms and foreign substances and avoids the development of destructive immune responses against invasive pathogens. The protective role of fish skin mucus is of immense economic significance, as infectious diseases limit intensive aquaculture globally.

In this study, the expression of 11 collagen-alpha members was extremely downregulated likely because of the increased production of parasite and carp proteases. It has been suggested that fish parasites release proteolytic enzymes to degrade collagen and other structural molecules to assist in disruption of external epithelia as an invasion strategy [19]. *I. multifiliis* proteolytic repertoire (degradome) includes 254 protease homologs, approximately 3.1% of the proteome [20].

Collagens are extracellular matrix proteins that play a structural role in the body of humans and animals. Collagen degradation and downregulation are believed to limit the peripheral damage of healthy tissues, first by releasing metalloproteinases to cleave collagen fibrils and then taking up the resulting fragments for wound healing. The responses because of skin damage signify a complex cascade of events that involves several overlapping stages including hemostasis, inflammation, proliferation, and maturation. The enzymes that destroy components of the extracellular matrix are involved in both inflammation and tissue repair and can be considered a bridge between these phases [21]. It has been reported that several matrix metalloproteinases are upregulated in Atlantic salmon skin during the early stages of infection by salmon louse [22]. These enzymes have a wide range of functions including the massive degradation of extracellular matrix and tissue remodeling to limit proteolysis and subtle regulation of immune processes [23]. In another study, cortisol and lice have been reported to equally downregulate a large number of motor proteins that have considerable roles in wound contraction and healing [21]. The downregulation of collagens and other structural proteins was in parallel with the induction of metalloproteinases that degrade extracellular matrix [21]. Cortisol and, to a lesser extent, lice enhanced the collagen-degrading matrix metalloproteinases. The down-expression of carp collagen proteins after exposure to *I. multifiliis* suggests a process of massive degradation of collagen aimed at tissue remodeling and wound healing of carp skin in a similar response pattern as for Atlantic salmon during the salmon louse infection experiments conducted by Krasnov et al. [21].

The expression of carp Src substrate cortactin-like protein was downregulated (1.2-fold) at 1 dpe but then increased (2.6-fold) at 9 dpe resulting in a considerable relative upregulation (3.3-fold). Cortactin regulates actin assembly and cell migration by stimulating actin-related protein Arp 2/3-mediated actin polymerization. High cortactin expression is associated with cell motility, invasion, and metastasis, and the elevated expression of cortactin correlates with the poor prognosis in human carcinomas [24]. Watt et al. [25] reported that protein tyrosine phosphorylation occurs within seconds of injury to the surface of intact articular cartilage, as does the activation of mitogen-activated protein kinases (MAPKs) and IκB kinase (IKK). The tyrosine-phosphorylated protein cortactin was identified together with adhesion kinase and paxillin as the substrates of Src family kinases. Injury to the surface of intact articular cartilage has been suggested to activate Src-like kinases, as well as MAPKs and IKK involved in NF-κB regulation [25]. The modulation of the carp Src substrate cortactin-like protein was likely to modulate NF-κB activation, aimed at reducing inflammation and tissue damage caused by *I. multifiliis*.

In this study, the expression of the carp dermatopontin-like protein was considerably downregulated (10.9-fold) at 9 dpe. Dermatopontin regulates the assembly of the extracellular matrix through the acceleration of collagen and fibronectin fibrillogenesis [26]. Recently, it has been suggested that it is involved in wound healing, particularly during re-epithelialization by promoting keratinocyte migration [27]. The considerable downregulation of the carp dermatopontin-like protein was likely to limit inflammation and tissue damage caused by *I. multifiliis*.

The expression of carp papilin protein was downregulated (11-fold) after exposure to *I. multifiliis*. Papilin interacts with several extracellular matrix components and enzymes and is essential for embryonic development of *Drosophila melanogaster* and *Caenorhabditis elegans* [28]. In vitro, papilin non-competitively inhibits procollagen N proteinase, an ADAMTS metalloproteinase. Targeted disruption of papilin by RNAi experiments on *Drosophila* embryos led to a repeated decrease in survival rates, correlated to muscle defects [28]. Thus, it has been suggested that papilin influences cell rearrangements in muscle tissue. The downregulation of the carp papilin was likely because of tissue damage as a result of the development and growth of *I. multifiliis*.

The expression of lumican protein was downregulated (ninefold) in infected carp. Lumican has several matricellular roles as an extracellular matrix constituent and as a matrikine involving in cell proliferation, gene expression, and wound healing [22]. Lumican regulates collagen fibrillogenesis, which is involved in the shaping and sustainment of clear corneas, promoting corneal epithelial wound healing and the integrity of numerous other connective tissues such as sclera, skin, and as a chemokine gradient maker [29]. In addition to regulating the collagen fibril architecture, lumican supports neutrophil recruitment and invasion after corneal damage and inflammation [30]. Lumican-deficient wounded mice corneas showed delayed healing, reduced recruitment of macrophages and neutrophils, and no induction of the proinflammatory cytokines, tumor necrosis factor-alpha (TNFα), and IL1β. Lumican is highly conserved between zebrafish and mammals, such as human and mouse, in respect to gene structure, expression patterns, and protein function [31]. Thus, the downregulation of lumican was likely to reduce inflammatory response and tissue damage caused by *I. multifiliis* and correlates with previous findings on fish gene expression mimicking tissue injuries and leukocyte recruitment [9].

In this study, carp keratin I cytoskeletal 18 protein was upregulated (11.4-fold) after *I. multifiliis* infection, while keratin I cytoskeletal 18-like was downregulated (3.5-fold) at 9 dpe. Keratin has been identified in the skin mucus of gilthead seabream [32], Atlantic cod [33], and European sea bass [1]. Keratin is a cytoskeletal protein whose primary function is to protect cells from mechanical and non-mechanical injuries. In addition, it has been shown that the keratin of fish mucus possesses antibacterial activity because of its pore-forming properties [34]. Keratin turnover is dependent on the ubiquitin–proteasome pathway, and its expression levels can be altered upon injury. Recently, it has been reported that the expression of keratin I and II was downregulated in cod mucus, following a vibrio infection, and in gilthead seabream in response to chronic wounds [1, 5]. The carp keratin I cytoskeletal 18 proteins were differentially regulated after *I. multifiliis* infection, suggesting they may play different sequential and/or site dependant roles in carp immune response aimed at protection against the parasite.

The expression of carp myosin proteins was considerably upregulated after *I. multifiliis* infection. In fish, disease resistance has been suggested to be associated with the expression of myosin heavy chain protein. The proteomic profiling of zebrafish fins was recently determined after a hemorrhagic septicemia *Rhabdovirus* infection using two-dimensional differential gel electrophoresis [35]. Several differentially expressed proteins identified were related to the cytoskeleton and involved in fin regeneration including myosin [35]. A gene expression study of Atlantic salmon (*Salmo salar*) showed differential expression of the transcripts of some proteins that have a major role in the transendothelial migration of leukocytes, including myosin during the early stages of infection by salmon louse [22]. The upregulation of the myosin proteins was likely to support the migration of leucocytes to the sites of infection as an active immune response of carp and to protect against tissue damage caused by *I. multifiliis*. The expression of carp MYH16 isoform X1 myosin protein was considerably upregulated (fourfold) at 1 dpe and then decreased (2.5-fold) at 9 dpe after *I. multifiliis* exposure, correlating with previously reported leukocyte recruitment patterns [9, 10].

In this study, the expression of neoverrucotoxin subunit beta protein was upregulated (3.5-fold) in the skin mucus of infected carp at 9 dpe. Genes that contain fibronectin type III and neoverrucotoxin subunit domains were predicted to be involved in microtubule organization and stabilization [36]. Neoverrucotoxin in fish venom has a mechanism of action where it has been shown to function by forming pores in cell membranes [37]. The skin toxins in Gobiodon have a range of biological functions, including parasite and predator prevention [38]. The upregulation of the neoverrucotoxin subunit beta protein was likely to defend fish against the parasite. However, its specific role in carp immune response against *I. multifiliis* should be thoroughly explored.

In this study, olfactomedin 4 was upregulated (3.3-fold) at 1 dpe but then was downregulated (1.3-fold) at 9 dpe. Olfactomedin 4 was originally detected in the preparations of chemosensory dendritic cilia obtained from olfactory epithelium of the bullfrog, *Litobathes catesbeianus* [39]. In addition, it is upregulated in inflammatory bowel diseases and *Helicobacter pylori*-infected patients. Olfactomedin 4 is the target gene of nuclear factor kappa B (NF-κB) pathway and has a negative feedback effect on NF-κB activation induced by *H. pylori* infection [40]. In a transcriptomic profiling study, several central signatures of catfish responses following columnaris infection were identified. A suppression of NF-κB signaling and olfactomedin 4 expression was observed in catfish gills, following columnaris infection [41]. Olfactomedin 4 expression was upregulated at 1 dpe and then reduced at 2 dpe. The expression pattern of olfactomedin 4 was similar to that observed in catfish gills, following columnaris infection as reported by Sun et al. [41], likely to reduce excessive tissue damage caused by carp inflammatory response induced by *I. multifiliis*.

Differentially regulated metabolism proteins were identified in carp skin mucus (Table 2). In this study, the carp ALOX5 proteins were downregulated. The lipoxygenases are lipid peroxidizing enzymes, which have been involved in the biosynthesis of pro- and anti-inflammatory mediators [42]. It transforms EFA substrates into leukotrienes. The overproduction of leukotrienes is a major cause of inflammation in asthma, allergic rhinitis, and osteoarthritis. In addition, they play a role in the pathogenesis of cardiovascular [43], hyperproliferative [44], and neurological [45] diseases. The finding that zebrafish (*Danio rerio*) expresses a functional ALOX5, together with the observation that most other human leukotriene-relevant genes have an ortholog in the zebrafish genome, suggests the biological relevance of leukotriene signaling in lower vertebrates [46]. The downregulation of the ALOX5 proteins was likely to reduce carp inflammatory response because of the invasion and development of *I. multifiliis*.

UDP-Glucose-6-phosphate dehydrogenase was overexpressed (4.4-fold) in the skin mucus of infected carp at 9 dpe. This protein catalyzes the conversion of glucose 6-phosphate to 6-phosphogluconate [47]. The most crucial function of this enzyme is the biosynthesis of glycosaminoglycans and other essential components of several extracellular matrix molecules, including chondroitin sulfate, heparan sulfate, and hyaluronic acid. The production of these molecules could affect several developmental processes. The changes in Glucose-6-phosphate dehydrogenase (G6PD) enzyme activity have been studied in liver and gill tissues of rainbow trout after exposure to organophosphate pesticide chlorpyrifos [48]. Acute exposure to chlorpyrifos exhibited apoptotic effects in the liver and gill tissues associated with a time-dependent decrease in G6PD enzyme activity at all concentrations tested [48]. The upregulation of G6PD likely activates tissue repair against damage caused by *I. multifiliis*.

The CLUH expression was upregulated during *I. multifiliis* infection and considerably increased (3.5-fold) at 9 dpe. Furthermore, CLUH is a cytosolic RNA-binding protein specific for a subset of mRNAs encoding mitochondrial proteins [49]. Thus, CLUH was considered as an excellent candidate for performing a post-transcriptional regulatory function. In HeLa cells, CLUH was reported to bind several mRNAs involved in intermediate metabolism and oxidative phosphorylation [49]. CLUH deficiency was found to affect respiratory function and mitochondrial DNA content, distribution, and ultrastructure [50]. The upregulation of CLUH protein expression apparently co-ordinates carp immune response to cope with adverse effects caused by this ciliate.

In this study, the expression of RAI protein was modulated after *I. multifiliis* infection. The expression of this protein was downregulated (1.2-fold) at 1 dpe, but then increased (2.8-fold) at 9 dpe resulting in a considerable relative upregulation (3.3-fold). RAI was reported to bind to the NF-κB subunit p65 and inhibit its transcriptional activity. RAI inhibited the action of NF-κB in a transient luciferase gene expression assay and the endogenous NF-κB activity induced by TNFα [51]. RAI can effectively block HIV-1 replication [52]. NF-κB is an inducible transcription factor in cells involved in immune and inflammatory responses. It induces the expression of cytokines, chemokines, and immunoreceptors [53]. Gonzalez et al. [9] identified two putative molecules of the NF-κB signaling pathway, which show strong similarity (63–68%) with the zebrafish IκBα in addition to a predicted molecule that is similar to NF-κB. It was the first time that molecules from the NF-κB signaling pathway were described in *I. multifiliis*-infected carp. The upregulation of the RAI protein expression likely inhibits NF-κB and decreases cytokine production to reduce inflammation and tissue damage induced by *I. multifiliis*.

PDZ/LIM domain 1-like protein was considerably upregulated (3.0-fold) at 9 dpe. The actin-associated protein family members containing PDZ/LIM protein–protein interaction motifs are involved in the heart and fin development of zebrafish [54]. In salmon, alpha-actinin-associated LIM protein containing PDZ and LIM motifs builds multi-protein complexes linking actin in muscles and non-muscular tissues [55]. PDLIM1, another member of LIM proteins, has been reported to negatively regulate NF-κB-mediated signaling in the cytoplasm. PDLIM1 sequestered the p65 subunit of NF-κB in the cytoplasm and suppressed its nuclear translocation in an IκBα-independent manner [56]. The upregulation of PDZ/LIM domain 1-like protein was likely because of the suppression of carp immune response to reduce inflammation and tissue damage caused by *I. multifiliis* attachment and growth. However, the specific role of this protein in carp skin mucus needs to be determined.

In this study, we identified proteins that were entirely novel in the context of the fish host response to *I. multifiliis*, such as olfactomedin 4, lumican, dermatopontin, papilin and I cytoskeletal 18. The modulation of these proteins in the fish skin mucus suggests a role in immune response aimed at protecting against tissue damage caused by this parasite. Proteases from parasitic protozoa have been involved in host invasion and emergence, encystment and excystment, cytoadherence, stimulation and evasion of host immune responses, and catabolism of host proteins for nutrients [57]. The cysteine protease, cathepsin L was differentially expressed among all life stages and was suggested to play important roles in host-pathogen interactions [58, 59]. Indeed, the largest up-regulation of cathepsin L cysteine protease was observed in the infective theront [59]. Hence, proteases from *I. multifiliis* have been suggested to suppress fish immune response, facilitate host invasion and support degradation of host cells [59]. We suggest that the degradation of collagens and the modulation of the other structural proteins lead to the downregulation of the inflammatory response by targeting the NF-κB pathway and binding to the NF-κB subunit p65 to inhibit its transcriptional activity aimed at reducing inflammation and protecting fish against tissue damage caused by *I. multifiliis*. However, specific, functional studies are required to support this suggestion and investigate the role of these differentially expressed proteins in tissue damage and wound healing to obtain a comprehensive understanding of the contributions of the fish mucus layer in defense against *I. multifiliis*. Using quantitative proteomics, the obtained results provide information on carp-*I. multifiliis* interactions and demonstrate proteomics as a non-invasive technique to give insights into the post-transcriptional and post-translational regulation of skin mucus proteins.

## Additional file

**Additional file 1.** **The STRING screenshot of supplied set of proteins involved in the protein-protein interaction network.** It shows details of protein abbreviation, node colour, edge interaction, network and functional enrichment: pathway and domain.
