# Supplementary figures and images for: Quantitative shotgun proteomics distinguishes wound-healing biomarker signatures in common carp skin mucus in response to Ichthyophthirius multifiliis
Source: Vet Res. 2018 Apr 20;49:37. doi: 10.1186/s13567-018-0535-9 (PMC5910588; doi:10.1186/s13567-018-0535-9)

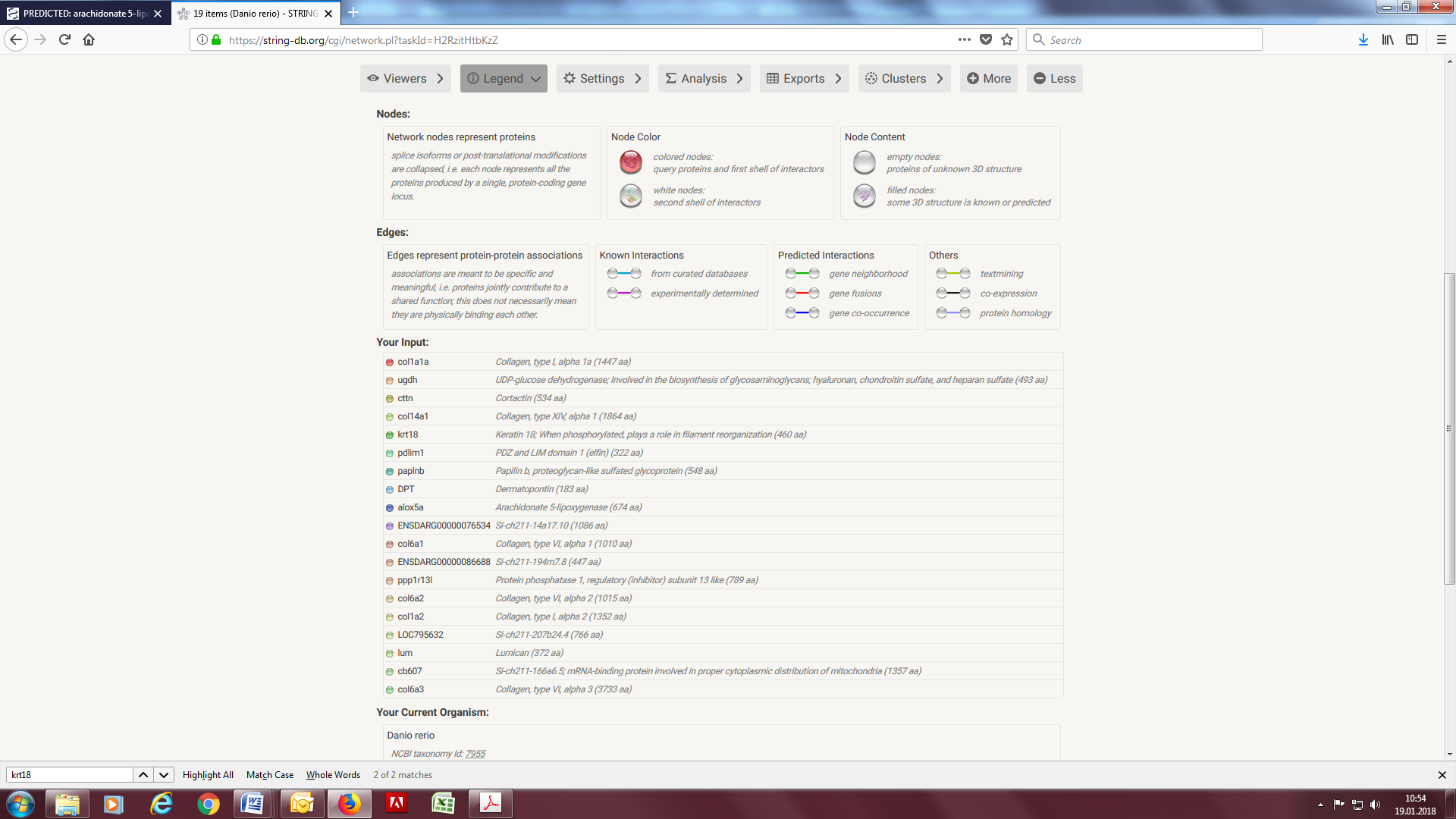


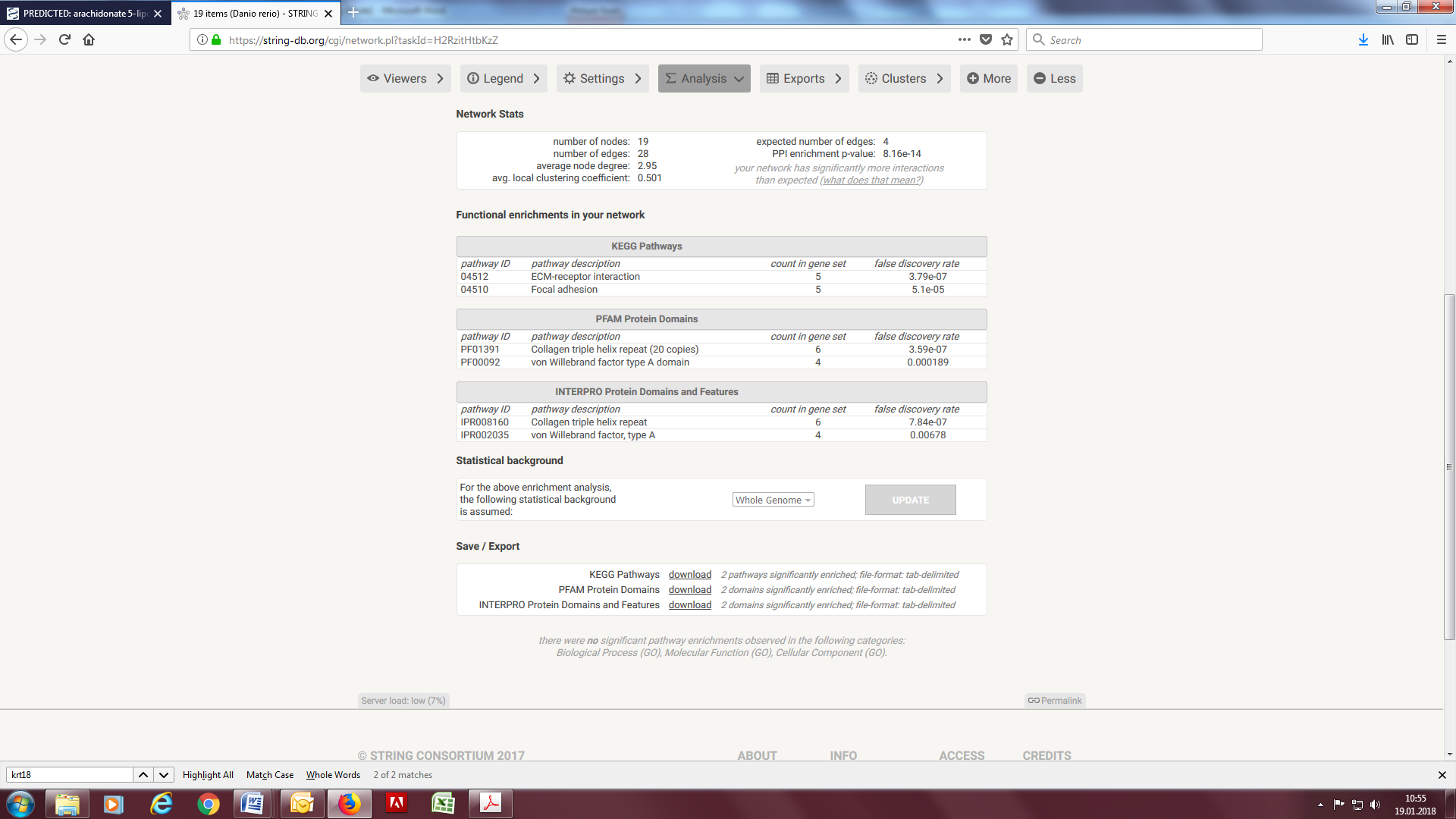

Supplement: Supplementary file 1 — Additional file 1. The STRING screenshot of supplied set of proteins involved in the protein-protein interaction network. It shows details of protein abbreviation, node colour, edge interaction, network and functional enrichment: pathway and domain. [file 13567_2018_535_MOESM1_ESM.doc]
